# Low serum levels of uric acid and albumin in patients with GuillainBarre syndrome: Erratum

**DOI:** 10.1097/MD.0000000000007318

**Published:** 2017-06-23

In the article, “Low serum levels of uric acid and albumin in patients with GuillainBarre syndrome”,^[1]^ which appeared in Volume 96, Issue 15 of *Medicine*, affiliations a, d, g, and h appeared incorrectly. They should have appeared as:

^a^ Department of Neurology, The First Affiliated Hospital of Wenzhou Medical University, Wenzhou

^d^ Department of Endocrinology, The First Affiliated Hospital of Wenzhou Medical University, Wenzhou

^g^ Department of Infection and Liver Diseases, Liver Research Center, The First Affiliated Hospital of Wenzhou Medical University, Wenzhou

^h^ Department of Cardiovascular Medicine, The Heart Center, The First Affiliated Hospital of Wenzhou Medical University, Wenzhou

## References

[1] Su Z Chen Z Xian Y Low serum levels of uric acid and albumin in patients with GuillainBarre syndrome. *Medicine*. 2017 96:e6618. 28403109 10.1097/MD.0000000000006618 PMC5403106

